# Effectiveness of Tomosynthesis Versus Digital Mammography in the Diagnosis of Suspicious Lesions for Breast Cancer in an Asymptomatic Population

**DOI:** 10.7759/cureus.13838

**Published:** 2021-03-11

**Authors:** Lourdes Noemi Santos Aragon, Dafne Soto-Trujillo

**Affiliations:** 1 Radiology, ABC Medical Center IAP, Mexico City, MEX

**Keywords:** breast cancer, digital breast tomosynthesis (dbt), re-staging

## Abstract

Introduction

The most frequent malignant tumor in women is breast cancer. A dense breast may mask lesions within the tissue. The constant improvement in diagnosis techniques has made the diagnosis more accurate. Digital mammography loses sensitivity in dense breasts as lesions may be masked by the over-position of tissue. Tomosynthesis increases sensitivity and specificity over diagnostic mammography. In this study, we examine the effectiveness of tomosynthesis versus digital mammography in asymptomatic patients.

Materials and methods

A cohort study of 1,499 Mexican patients that came for screening at a private health service from January to December 2015. A Breast Imaging Reporting and Database System (BI-RADS) classification was given by a breast radiologist with the digital mammography reading. Later, a second breast radiologist reviewed the same patients with tomosynthesis and assigned a second BI-RADS category.

Results

Patients were divided into three age groups. The one with the most had patients between 40-49 years (51.3%), where re-staging to a higher BI-RADS occurred in 40 patients. Re-staging to a lower category was most common in the group of age above 50, where 30 patients were assigned BI-RADS 2 after tomosynthesis. Dense breast (C and D) represented 38%. After tomosynthesis, 28 patients were classified as BI-RADS 4 or 5. The prevalence of diseases in groups BI-RADS 4 and BI-RADS 5 after re-staging and a breast cancer result was 0.024, with a sensitivity of 54% and a specificity of 88%. When re-staging 2D mammography with 3D tomosynthesis for suspicious lesions classified BI-RADS 3, 4, or 5, the prevalence was 0.23, with a sensitivity of 45% and a specificity of 98%.

In this study, patients were asymptomatic, yet 20 breast cancers were detected, with a sensitivity of 54% and a specificity of 88%, exceeding the specificity of diagnostic mammography. Moreover, when re-staging to a BI-RADS of suspicious findings, the sensitivity was 45%, with a specificity of as high as 98%.

## Introduction

Breast cancer is the most frequent malignant tumor in women in the world; due to this, there is a need to implement measures that improve treatment and the diagnosis so they are effective and timely.

The overall global mortality for breast cancer is 13.6 and the incidence is 47.8 per 100,000. In 2020, there were 2,261,419 new cases reported (representing 11.7% of all new cancers), with mortality of 684,996 women. The five-year prevalence is 7,790,717 women [1].

In Mexico, the incidence in 2020 was 28.2 per 100,000 women, with a five-year prevalence of 99,288 women. Breast cancer is the leading cause of cancer in women, followed by cervical cancer, with an incidence of 8.9 per 100,000 [2].

The average age of presentation of breast cancer is 54.9 years, with the highest incidence in the age group of 50 to 59 years, which has 45% of all cases [2]. 

The accurate diagnosis of breast cancer with breast imaging studies is vital to personalize the strategies for detecting cancer [3]. Strategies are being created around the world to improve the diagnosis and treatment to improve survival. There are imaging methods that are auxiliary in diagnosing breast cancer and screening programs that are part of programs used worldwide as part of a timely diagnosis.

Mammography is the only study for screening in asymptomatic patients, and it is the initial imaging study in the diagnosis of symptomatic patients, with a sensibility for suspicious lesions of 86% and a specificity of 57%. Additional imaging tools are emerging to detect suspicious lesions that improve the diagnosis, such as tomosynthesis, which has a diagnostic sensitivity of 93% and a specificity of 70%. In symptomatic women, it has shown superiority compared to conventional mammography (2D).

Digital breast tomosynthesis (DBT) is a novel imaging technology that enables the production of three-dimensional volumetric images (3D), which reduces tissue overlap and increases breast cancer detection sensitivity and specificity. Images are obtained in the usual mammography projections in mediolateral oblique (MLO) view and a craniocaudal (CC) view for each breast. The X-ray tube has a positive and negative movement of 15 to 50 degrees, depending on the degree of displacement that the mammary gland's thickness. The acquisition time, which depends on the mammary gland’s morphologic characteristics, can range from 10 to 25 seconds for each projection. The result varies from 11 to 19 images, which allow a reconstruction of 1 mm. The reader scrolls through individual or multiple sections. Artifacts can mimic or obscure these pathologic changes and reduce the sensitivity or specificity of the modality [4]

DBT is more available as a development of digital mammography. Its additional or substitutive application is hoped to further increase the accuracy of imaging assessment. As Heywang-Köbrunner [5] reported, the use of DBT for lesion assessment reduces both histopathological assessments and short-term follow-up examinations, as well as patient distress and costs, while improving diagnostic accuracy; and when comparing with additional mammographic views, the overall radiation dose of assessment with DBT would remain more or less unchanged compared with radiation with additional mammographic views. 

In radiology, suspicious lesions in the mammary gland are characterized based on specific findings based on criteria that guide the clinician to make essential decisions for the patient. This characterization is based on the Breast Imaging Report and Data System (BI-RADS) fifth edition of the American College of Radiology [6].

Lesions that are considered by mammography as suspicious include nodules based on their margins, form, and density; microcalcifications based on their distribution of form - if they are amorphous, heterogeneous, fine pleomorphic, or linear branched; architectural distortions when there is no history of surgical procedure and asymmetries that do not modify or appear for the first time.

In the BI-RADS report, breast density, radiological findings, location, and follow-up behavior are reported. All these aspects are stated in a conclusion, thus allowing all those involved in the patient's attention, diagnosis, and treatment to use the same terminology and behavior. With these criteria, the radiological diagnosis recommended by the American College of Radiology is established, which can go from 0 to 6 based on the findings:

0: Need of additional imaging or prior examinations

1: Normal

2: Benign findings

3: Probably benign findings that require a short-interval follow-up. Positive predictive value (PPV) <2%

4a: Suspicious finding, tissue diagnosis is recommended. PPV 2-10%

4b: Suspicious finding, tissue diagnosis is recommended. PPV 10-50 %

4c: Suspicious finding, tissue diagnosis is recommended. PPV 50-95%

5: Suspicious finding, tissue diagnosis is recommended. PPV > 95%

6: Known biopsy-proven malignancy

Breast tissue is visually classified into four groups according to its composition:

Type A: The breast is almost entirely fatty

Type B: There are scattered areas of fibroglandular density

Type C: The breast is heterogeneously dense, which may obscure small masses

Type D: The breast is extremely dense, which lowers the sensitivity of mammography.

Tomosynthesis

The indications are not yet well established regarding the application of tomosynthesis. It has been concluded that its most significant value is achieved when characterizing a specific lesion within a dense breast.

In the re-staging of suspected lesions, tomosynthesis has superiority in lesions categorized as BI-RADS 3, such as an architectural distortion in patients with surgical history, since cancer cannot be excluded with certainty, or in patients where a benign diagnosis is given when fat necrosis is beginning to form. A radiological diagnosis is complicated, and correct identification is essential because of the high PPV for cancer up to 60% [7]. Its detection is difficult in 2D mammography since the superimposition of fibroglandular tissue in dense breasts.

Of the cancers that manifest as architectural distortion in tomosynthesis, 50% are usually hidden in 2D digital mammography and 20% are categorized as asymmetries using the same imaging method [8]. Most of the cancers diagnosed that are associated with architectural distortion correspond in histopathology to the lobular subtype.

In most cases, suspicious lesions in 2D tomosynthesis correspond to tissue overlapping. With the use of tomosynthesis, recategorization at the moment is done. This prevents unnecessary follow-ups and biopsy and speeds up the diagnosis for lesions categorized as BI-RADS 4 with a quick realization of a biopsy and histopathological diagnosis [9].

Nodules are better characterized with tomosynthesis since they avoid using ultrasound or give a better overview of the surrounding tissue before performing the ultrasound. When a nodule is visualized in a dense breast, the edges or part of them may be hidden in the surrounding tissue. Tomosynthesis allows determining the tissue over position and edges of the nodule. Nodules may have malignancy characteristics such as irregular or spiculated margins that were not first seen with conventional mammography. Also, when measuring a mass with tomosynthesis, it is possible to discern the spicules of the mass, which allows a measurement closer to reality and thus a correct measurement in patients where a response to oncological treatment is evaluated [10].

Another use of tomosynthesis is to provide a visual guide for interventional procedures, such as localization and biopsies. Besides, it also serves to evaluate multifocal or multicentric disease as well as for surgical planning due to its 3D orientation for both radiologist and surgeon for planning because it provides the depth of the lesion [11]. In some specific cases, it is even substituting the use of MRI.

New technologies such as tomosynthesis make it possible to solve tissue superimposition [12] since it is a tool that allows the acquisition of images in three dimensions and thus improves the evaluation of lesions, their margins, shape, and associated findings such as asymmetry of architectural distortion. Another advantage is reducing additional projections needed for diagnosis, such as lateral projections or magnifications, among others [13].

Few studies determine the usefulness in the screening of tomosynthesis regardless of the type of breast tissue and other risk factors already established for breast cancer. The objective of the study is to demonstrate the usefulness of tomosynthesis compared to digital mammography in asymptomatic women.

## Materials and methods

Study design and data source

A historical cohort study of Mexican patients that were classified according to the BI-RADS classification of the American College of Radiology in asymptomatic women of private health services in Mexico was analyzed. Data was provided by an electronic file of the hospital. This analysis was done in all cases registered in this dataset up until January to December 2015, with a total of 1,499 patients. All patients had standard CC and MLO projections and subsequently, digital breast mammography using the Selenia Dimensions Mammography System (Hologic, Inc., Marlborough, MA, USA) was performed in the same projections. 

Variable definition

Our dataset includes demographic characteristics such as age, hereditary family history, hormone replacement therapy, surgical history in the breast, and type of breast tissue.

Patients were divided into three age groups: under 40, 40 to 49, and over 50 years. The hereditary family history, history of hormone replacement therapy, and surgical history created dichotomous variables. For the type of breast tissue, we considered the American College of Radiology classification of A, B, C, or D.

First, the breast radiologist's radiological diagnosis of the screening mammogram was recorded; it was defined as initial BI-RADS. A second breast radiologist then reviewed the same group of patients with tomosynthesis and established an assigned BI-RADS for a second time.

Statistical analysis

Demographic features and BI-RADS were compared between two categories. Data was analyzed with McNemar's test as they are related variables. The rest of the variables were checked with Chi-squared test analysis. Afterward, we performed a bivariate analysis comparing demographic characteristics and re-staging BI-RADS using tomosynthesis using the Chi-squared test.

The logistic regression model is presented with the Odds Ratio (OR) and its respective 95% Confidence Interval (CI 95%). Statistical significance was set at p<0.05 and performed with SPSS version 25.0 (IBM Corp., Armonk, NY, USA).

## Results

A total of 1,449 women were studied. The first group categorized women under 40 years with 154 women (10.3%). In the second group, women between 40 and 49 were included, with 769 women (51.3%). The third group comprised women of 50 years or older, with 576 women (38.4%).

When re-staging to a higher BI-RADS classification after tomosynthesis, in the first group of under 40, 126 women initially had BI-RADS 3, and after tomosynthesis 137 women were BI-RADS 3. In this same age group, three women were classified initially as BI-RADS 4 and later eight.

In the second age group of 40 to 49 years, the change to BI-RADS 2 was from 614 with mammography to 654 with tomosynthesis. BI-RADS 4 went from 27 to 45 women after re-staging. A restaging to a BI-RADS 5 occurred from one initial patient to five after tomosynthesis in this group.

Finally, in the age group of 50 years or older, more restaging to a lower BI-RADS was observed, where 481 women were initially given BI-RADS 2 and after tomosynthesis, 511 were classified as benign findings. Moreover, 16 women were initially given BI-RADS 3, and after tomosynthesis, 35 were part of this classification.

When considering positive family history, re-staging happened in 170 women classified as BI-RADS 2 initially, and after tomosynthesis, 192 women were BI-RADS 2. In suspicious findings given BI-RADS 4, 20 women were initially classified, and after an evaluation with tomosynthesis, 33 were re-staged. In women with a history of hormone replacement therapy, BI-RADS 4 classification went from 12 to 42 women. A history of a surgical history had similar results since the group ages presented similar results where BI-RADS 2 category went from 366 women to 414 and BI-RADS 4 went from 10 to 4.

Based on tissue density, groups C and D are considered dense, and this subgroup represented 569 patients (38%). In non-dense breast (types A and B), re-staging from BI-RADS 2 and BI-RADS 3 went from 860 to 880 women, and re-staging to BI-RADS 4 and BI-RADS 5 went from 21 to 32 women.

In dense breasts, 498 initially were given BI-RADS 2 and BI-RADS 3, and later, 514 after tomosynthesis. Furthermore, BI-RADS 4 and 5 went from 24 to 52 patients (Table 1).

**Table 1:** Demographic characteristics according to the initial BI-RADS with mammography and later BI-RADS with tomosynthesis *Type of breast tissue: A, adipose; B, scattered fibroglandular; C, heterogeneously dense; D, extremely dense. BI-RADS: Breast Imaging Reporting and Database System.

		Initial BI-RADS												
		0		1		2		3		4		5		
		N %		N %		N %		N %		N %		N %		P value
Age group (years)	<40y.	7	4.5	2	1.3	126	81.8	16	10.4	3	1.9	0	0	0.343
	40-49	49	6.4	12	1.6	614	79.8	66	8.6	27	3.5	1	0.1	
	>49y.	18	3.1	8	1.4	481	83.5	55	9.5	14	2.4	0	0	
		Later BI-RADS												
	<40y.	0	0	0	0	137	89.0	9	5.8	8	5.2	0	0	0.525
	40-49	0	0	9	1.2	654	85.0	56	7.3	45	5.9	5	0.7	
	>49y.	0	0	4	0.7	511	88.7	35	6.1	23	4.0	3	0.5	
Hereditary family history		Initial BI-RADS												
	yes	21	8.7	2	0.8	170	70.5	28	11.6	20	8.3	0	0	<0.001
	no	53	4.2	20	1.6	1551	83.5	109	8.7	24	1.9	1	0.1	
		Later BI-RADS												
	yes	0	0	0	0	192	79.7	16	6.6	33	13.7	0	0	<0.001
	no	0	0	13	0.1	1110	88.2	84	6.7	43	3.4	8	0.6	
Hormone replacement therapy		Initial BI-RADS												
	yes	8	4.4	0	0	138	76.7	21	11.7	12	6.7	1	0.6	<0.001
	no	66	5.0	22	1.7	1083	82.1	116	8.8	32	2.4	0	0	
		Later BI-RADS												
	yes	0	0	0	0	134	74.4	4	2.2	42	23.3	0	0	<0.001
	no	0	0	13	1.0	1168	88.6	96	7.3	34	2.6	9	0.6	
Surgical history in breast		Initial BI-RADS												
	yes	18	3.8	21	4.4	366	76.7	62	13.0	10	2.1	0	0	<0.001
	no	56	5.5	1	0.1	855	83.7	75	7.3	34	3.3	1	0.1	
		Later BI-RADS												
	yes	0	0	13	2.7	414	86.8	46	9.6	4	0.8	0	0	<0.001
	no	0	0	0	0	888	86.9	54	5.3	72	7.0	8	0.8	
Type of breast tissue*		Initial BI-RADS												
	A	7	4.7	5	3.4	127	85.8	7	4.7	2	1.4	0	0	0.001
	B	27	3.5	10	1.3	657	84.0	69	8.8	19	2.4	0	0	
	C	33	6.3	7	1.3	405	77.0	57	10.8	23	4.4	1	0.2	
	D	7	16.3	0	0	32	74.4	4	9.3	0	0	0	0	
		Later BI-RADS												
	A	0	0	4	2.7	138	93.2	2	1.4	3	2.0	1	0.7	<0.001
	B	0	0	6	0.8	713	91.2	35	4.5	24	3.1	4	0.5	
	C	0	0	3	0.6	416	79.1	59	11.2	45	8.6	3	0.6	
	D	0	0	0	0	35	81.4	4	9.3	4	9.3	0	0	

Among women who were re-staged to an upper BI-RADS category, such as 4 or 5 with suspicious lesions, and underwent image-guided biopsy with thick needle in this study, 20 had cancer. Infiltrating ductal carcinoma was the most common type. Two patients had ductal hyperplasia with atypia. 

The prevalence of disease in groups BI-RADS 4 and BI-RADS 5 after re-staging and a breast cancer result was 0.024, with a sensitivity of 54% and a specificity of 88%.

When re-staging 2D mammography with 3D tomosynthesis for suspicious lesions classified as BI-RADS 3, 4, or 5, the prevalence was 0.23, with a sensitivity of 45% and a specificity of 98% (Tables 2, 3).

**Table 2:** Breast cancer and re-staging of BI-RADS with tomosynthesis in a 2 x 2 table Prevalence: 0.024, sensitivity: 54%, specificity: 88% BI-RADS: Breast Imaging Reporting and Database System

	Breast Cancer		
Re-staging	Yes	No	Total
Yes	20	164	184
No	17	1298	1315
Total	37	1462	1499

**Table 3:** Re-staging with Tomosynthesis and Assignment of Suspected BI-RADS (3, 4, and 5) Prevalence: 0.23, sensitivity: 45%, specificity: 98% BI-RADS: Breast Imaging Reporting and Database System

	Re-staging with Tomosynthesis		
Suspected BI-RADS	Yes	No	Total
Yes	163	21	184
No	194	1121	1315
Total	357	1142	1499

In the bivariate model, the variables with significant results, and which are a risk for the patient at the time of re-staging BI-RADS with the use of tomosynthesis, are positive family history with an OR of 2.3 (95% CI 1.7-3.2) p<0.001, use of hormone replacement therapy OR 3.6 (CI 95% 2.6-5.0) p<0.001, and tissue breast density OR of 1.6 (95% CI 1.3-1.9) p<0.001.

Age and a prior surgery history were not significant in this model with an OR of 0.8 and 0.9 (CI 95% 0.6-1.0 and 0.7-1.2 for each variable) (Table 4).

**Table 4:** Bivariate model of re-staging with tomosynthesis. OR, odds ratio; CI, confidence interval

	OR	CI 95%	P-value
Age, over 50 years	0.8	0.6- 1.0	0.061
Hereditary family history	2.3	1.7-3.2	<0.001
Hormone replacement therapy	3.6	2.6-5.0	<0.001
Surgical history in breast	0.9	0.7-1.2	0.561
Type of breast tissue	1.6	1.3-1.9	<0.001

The suspicious findings that were characterized by tomosynthesis were asymmetries in 28.5% of the cases (OR 1.3; CI 95% 0.98-1.8; p=0.109), architectural distortion 58.8% (OR 4.8; CI 95% 2.7-8.6; p<0.001), microcalcifications 80% (OR 14.4; CI 95% 7.3-28.3; p<0.001), and nodules 53.1% (OR 5.0; CI 95% 3.8-6.8; p<0.001).

The significant findings for a suspicious diagnosis for malignancy were architectural distortion, microcalcifications, and a nodule (Table 5).

**Table 5:** Re-staging with tomosynthesis and findings found OR, odds ratio; CI, confidence interval

	Re-staging with tomosynthesis			
	Frequency	OR	CI 95%	P-value
Asymmetry	53 (28.5%)	1.3	0.98 - 1.8	0.109
Distortion of architecture	30 (58.8%)	4.8	2.7 - 8.6	<0.001
Microcalcifications	44 (80%)	14.4	7.3 - 28.3	<0.001
Nodule	128 (53.1%)	5.0	3.8 - 6.8	<0.001

**Figure 1:**
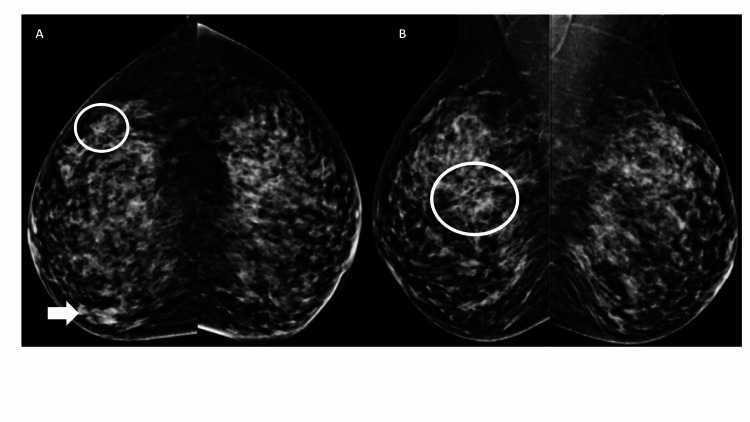
In the initial digital mammography CC (A) and MLO (B) projections show an architectural distortion in the external upper quadrant of the right breast (circles) CC, craniocaudal; MLO, mediolateral oblique

The case of a 54-year-old female patient who came to screening mammography is presented next. She had no prior history of breast cancer in her family, surgery, or use of hormone replacement therapy (Figure 1). 

**Figure 2:**
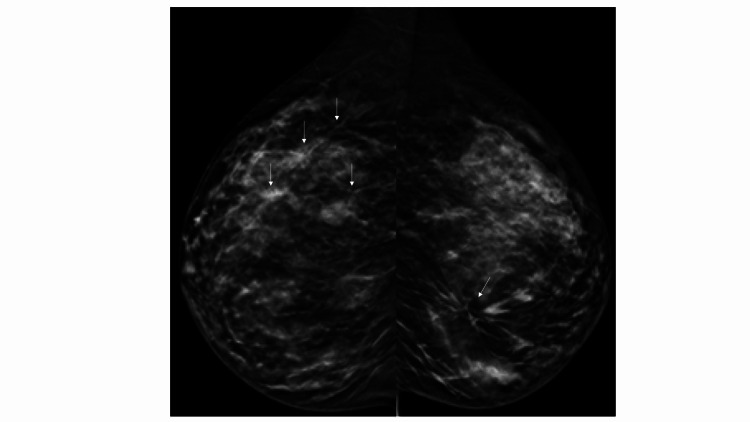
With tomosynthesis, four architectural distortions in the right breast and one in the left breast were evident (arrows)

Tomosynthesis revealed architectural distortions not evident with digital mammography (Figure 2).

## Discussion

Tomosynthesis allows for a more accurate diagnosis for the patient at the time of acquisition of the images. The X-ray equipment acquires the images in a single moment, and the programs perform 2D and 3D without the need to obtain the two studies separately with the usual mediolateral oblique (MLO) and craniocaudal (CC) projections.

In this study of 1,499 women, for the group at most significant risk of breast cancer by age, women aged 50 or older, tomosynthesis could re-stage to a lower BI-RADS category, thus avoiding unnecessary follow-up that distress patients and generate more expenses.

Family history of breast cancer and use of hormone replacement therapy are important variables for breast cancer risk in benign and suspicious findings because of their impact in re-staging, and they are considered a risk for breast cancer demonstrable in this study.

An important consideration is a dense breast that has been shown to be an independent risk factor for breast cancer [14]. Breast density is a radiological feature that can be observed in mammography and a dense breast differs from a non-dense breast tissue since it has areas of higher density, which may mask lesions. Up to 27% of breast cancers are missed in a dense breast [15]. With tomosynthesis, this disadvantage of 2D mammography is reduced. In this study, it was observed that 1,020 patients with dense breast tissue were assigned to a lower BI-RADS category of benign findings when asymmetries were first identified. In the case of architectural distortions, microcalcifications, and nodules, they were re-staged to a suspicious category with the indication of biopsy for risky lesions.

Tomosynthesis increases sensitivity and specificity over diagnostic mammography, which is when a patient presents with a symptom such as a palpable nodule, pain, skin changes, or nipple discharge. Tomosynthesis has a sensitivity of 93% and a specificity of 70%. In this study, patients were asymptomatic, yet 20 breast cancers were detected, with a sensitivity of 54% and a specificity of 88%, exceeding the specificity of diagnostic mammography. Moreover, when re-staging to a BI-RADS of suspicious findings, the sensitivity was 45% with a specificity of as high as 98%.

The implementation of tomosynthesis in asymptomatic women would imply, as in the paper of Hofvin [16], that tomosynthesis is profitable for all the benefits already discussed in this study which result in saving in the number of projections, follow-ups, and complements, furthermore, if we add that the radiation dose with single-display acquisition systems is reduced by obtaining a single acquisition with 2D and 3D images. Radiation will no longer be an inconvenience when using tomosynthesis for patients [17].

## Conclusions

Tomosynthesis is an effective imaging method in the assessment of breasts with heterogeneously dense and extremely dense breasts in re-staging to a higher or lowers BI-RADS, avoiding additional projections at the time of screening, and helping characterize architectural distortion-type lesions that in reality correspond to over position of tissue or post-surgery related changes. When microcalcifications are detected, it helps determine dispersion or grouping, and in nodules, it improves the determination of margins and density. It also helps to diagnose suspicious lesions hidden with tissue overlap and not seen in 2D mammography, which allows convenient biopsies and a definite diagnosis.
